# Supplementary material for: Morphometry of cellular behavior of coelomocytes from starfish Asterias amurensis
Source: PeerJ. 2021 Nov 23;9:e12514. doi: 10.7717/peerj.12514 (PMC8621724; doi:10.7717/peerj.12514)
Supplement: Supplemental Information 1 [file peerj-09-12514-s001.docx]

Supplemental 1.

Linear parameters:

*Area*;

*Circularity*: 4π*area / perimeter^2^, (***Circ***);

*Feret's Diameter* –- the longest distance between any two points along the object boundary, also known as maximum caliper, (***Feret***);

*Aspect ratio*: major_axis/minor_axis of object, (***AR***);

*Roundness*: 4*area /(π*major_axis^2^), (***Round***);

*Density*=Foreground Pixels/Hull Area, (***Density***);

*Hull's Perimeter* – perimeter of the convex hull drawn around the object, (***Hull'sPer***);

*Hull's Circularity* – Circularity of the convex hull = 4π*area ⁄ perimeter^2^, (***Hull'sCirc***);

*Max/Min Radii from Hull's Centre of Mass* – the ratio of maximum and minimum radii from the centre of mass for the convex hull to an exterior point, (***M/MHull'sCM***);

*Mean Hull's Radius* – The mean length from the centre of mass of the convex hull to an exterior point, (***MeanHull'sRad***);

*Diameter of Bounding Circle* – bounding circle is calculated using the maximum span across or else the three points defining the smallest circle around the convex hull, (***DiamBoundCirc***);

*Max/Min Radii from Circle's Centre* – The ratio between the maximum and the minimum radii from the circle's centre to a point on the convex hull, (***M/M RadCirc***);

*Mean Radius from Circle's Centre* – The mean radius from the circle's centre to the points of the convex hull, (***MeanRadCirc***);

The ratio of area parts of the cell that turned out to be in the two halves of the bounding circle, the diameter dividing cells was drawn in the direction that most unevenly divided the image cells and was used as a measure of cell asymmetry, (***1/2half***);

The ratio of cell area in the outer half of the bounding circle to the area of ​​the cells in the inner half of bounding circle, (***in50/out50***);

*Perimeter* of cell, (***Per***);

*Roundness* of the outline picture of cells, (***Round out***).

Nonlinear parameters:

*Mean D* =Σ(D)/GRIDS (D=slope (ln(Boxes with Foreground Pixels)/ln(ε)), where ε - [box size](http://rsbweb.nih.gov/ij/plugins/fraclac/FLHelp/Glossary.htm#boxsize) or [scale](http://rsbweb.nih.gov/ij/plugins/fraclac/FLHelp/Glossary.htm#epsilon), the average D_B_ (box-counting fractal dimension) from multiple box counting scans, each delivering its own D_B_, based on a different orientation in the same image of the same series of grid calibers, (***MeanD***);

Fractal dimension of contour images of cells calculated using the method described above, (***outMeanD***);

*Mean ΛD* – The lacunarity based box counting dimension (ΛD_B_) or its average ΛD_B_ = (Grids∑G=1(ΛD_B(G)_))×Grids^-1^ , where ΛD_B(G)_ is the lacunarity calculated for each orientation of the grid, Grids are all possible orientations of the grid, (***MeanΛD***);

Lacunarity of contour images of cells calculated as described above, (***outMeanΛD***);

*Mean Smoothed (Biggest) D* – D_B_ calculated with allowance only for the squares larger than the average size, (***BiggestD***);

BiggestD of contour images of cells, calculated using the method described above, (***outBiggestD***);

*Mean Smoothed (Smallest) D* – (Smoothed D_B(small)_) – D_B_ calculated with allowance only for the squares smaller than the average size, which assumes that increases in count with increases in size should be ignored and that the smallest possible box for a given count holds density most efficiently, (***SmallestD***);

Smallest D of contour images of cells calculated using the method described above, (***outSmallestD***);

Lacunarity L(F (foreground mass))=(Σ(FΛ))/GRIDS – lacunarity based on the variation in pixels per box summarized over all grid orientations for an image, "F" refers to foreground pixels per box = (Σ(FΛ))/GRIDS, (***LF***);

Lacunarity L(E (foreground and empty space))=(Σ(EΛ))/GRIDS – lacunarity calculated on the basis of differences in the number of pixels in each square of the grid for all orientations of the squares, with allowance for the background pixels taken as zero (only pixels of the image of the object were taken into account in LF), (***LE***);

*Variation in Count*=Σ(Count(σ/μ))/GRIDS – The relative variation in the number of boxes it took to cover an image for the mean D_B_ over all grid orientations, (***Var in Count***);

Average lacunarity calculated for different orientations of the square grid, (***MeanΛ***);

*Mean Local Connected Fractal Dimension* – connected set is that all foreground pixels that are in the 8x8 environment of a seed pixel are considered connected, and this basic rule is applied to find the connected set for some predetermined arbitrary distance around that starting pixel, (***MeanLCFD***);

The average dimension of contour images of cells, calculated as described above, (***outMeanLCFD***);

*LCFD Prefactor Lacunarity* – a measure of heterogeneity or translational invariance dependent on where a grid series is placed, Local Connected Fractal Dimension is found from a type of fractal analysis that uses pixel mass from concentrically placed sampling units, using the connected set at each pixel to produce a distribution of local variation in complexity. Prefactor lacunarity is lacunarity assessing by using box counting, the Prefactor method, is based on the value obtained from box counting for the fractal dimension (
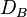
). This statistic uses the variable 
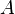
 from the scaling rule 
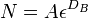
, where 
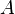
 is calculated from the y-intercept (
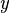
) of the ln-ln regression line for 
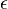
 and either the count (
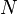
) of boxes that had any pixels at all in them or else 
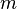
 at 
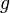
. 
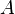
 is particularly affected by image size and the way data are gathered, (***LCFD PreLac***);

A measure of heterogeneity or translational invariance dependent on where a grid series is placed calculated for contour images of cells, (***outLCFD PreLac***);

*Mean Local Fractal Dimension* – the average dimension calculated on the basis of multiple [sampling](http://rsbweb.nih.gov/ij/plugins/fraclac/FLHelp/Glossary.htm#scan) an image randomly or systematically and defining the local fractal dimension of various parts of the image, (***MeanLFD***);

Mean Local Fractal Dimension of contour images of cells calculated as described above, (***outMeanLFD***);

*Mean Mass Fractal Dimension* – the [D_B_](http://rsbweb.nih.gov/ij/plugins/fraclac/FLHelp/Glossary.htm#db) calculated using [pixels per box](http://rsbweb.nih.gov/ij/plugins/fraclac/FLHelp/Glossary.htm#ppb), D_Bmass_= limε→0(^ln μ^_ε_ ⁄_ln ε_), limε→0 is found as the [slope of the regression line](http://rsbweb.nih.gov/ij/plugins/fraclac/FLHelp/Calculations.htm#regression) for μ_ε_ and ε, μ_ε_ = the mean [pixels per box](http://rsbweb.nih.gov/ij/plugins/fraclac/FLHelp/Glossary.htm#ppb) at some ε, where ε = [box size](http://rsbweb.nih.gov/ij/plugins/fraclac/FLHelp/Glossary.htm#boxsize) or [scale](http://rsbweb.nih.gov/ij/plugins/fraclac/FLHelp/Glossary.htm#epsilon), (***MMFD***);

Mean mass dimension of contour images of cells calculated as described above (***outMMFD***).
